# 1-(2-Bromophenyl)ethane-1,2-diyl 1,1′-biphenyl-2,2′-dicarboxylate

**DOI:** 10.1107/S1600536812018053

**Published:** 2012-04-28

**Authors:** Hoong-Kun Fun, Ching Kheng Quah, Dongdong Wu

**Affiliations:** a X-ray Crystallography Unit, School of Physics, Universiti Sains Malaysia, 11800 USM, Penang, Malaysia; b School of Chemistry and Chemical Engineering, Nanjing University, Nanjing, 210093, People’s Republic of China

## Abstract

In the title compound, C_22_H_15_BrO_4_, the bromo­benzene ring is inclined at dihedral angles of 23.87 (11) and 52.37 (11)° with respect to the planes of the two benzene rings. The two benzene rings of the biphenyl unit form a dihedral angle of 49.08 (11)°. In the crystal, mol­ecules are linked into [100] chains by C—H⋯O hydrogen bonds.

## Related literature

For a related structure, references to other similar structures and chemical and biological background, see: Fun *et al.* (2012) ▶. For the preparation, see: Wu *et al.* (2012) ▶. 
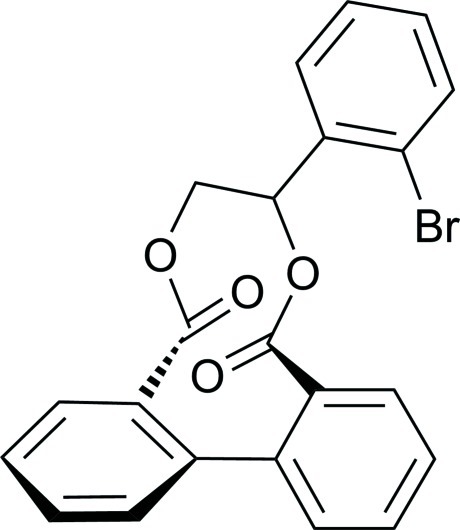


## Experimental

### 

#### Crystal data

C_22_H_15_BrO_4_
*M* *_r_* = 423.25
Monoclinic, 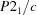

*a* = 8.0436 (9) Å
*b* = 21.775 (2) Å
*c* = 12.8809 (11) Å
β = 125.613 (5)°
*V* = 1834.1 (3) Å^3^
*Z* = 4
Mo *K*α radiation
μ = 2.27 mm^−1^
*T* = 296 K
0.41 × 0.23 × 0.17 mm

#### Data collection

Bruker SMART APEXII DUO CCD diffractometer
Absorption correction: multi-scan (*SADABS*; Bruker, 2009 ▶) *T*_min_ = 0.456, *T*_max_ = 0.702
17934 measured reflections
5372 independent reflections
3377 reflections with *I* > 2σ(*I*)
*R*_int_ = 0.028

#### Refinement

*R*[*F* ^2^ > 2σ(*F* ^2^)] = 0.039
*wR*(*F* ^2^) = 0.102
*S* = 1.02
5372 reflections
244 parameters
H-atom parameters constrained
Δρ_max_ = 0.50 e Å^−3^
Δρ_min_ = −0.64 e Å^−3^

### 

Data collection: *APEX2* (Bruker, 2009 ▶); cell refinement: *SAINT* (Bruker, 2009 ▶); data reduction: *SAINT*; program(s) used to solve structure: *SHELXTL* (Sheldrick, 2008 ▶); program(s) used to refine structure: *SHELXTL*; molecular graphics: *SHELXTL*; software used to prepare material for publication: *SHELXTL* and *PLATON* (Spek, 2009 ▶).

## Supplementary Material

Crystal structure: contains datablock(s) global, I. DOI: 10.1107/S1600536812018053/hb6749sup1.cif

Structure factors: contains datablock(s) I. DOI: 10.1107/S1600536812018053/hb6749Isup2.hkl

Supplementary material file. DOI: 10.1107/S1600536812018053/hb6749Isup3.cml

Additional supplementary materials:  crystallographic information; 3D view; checkCIF report

## Figures and Tables

**Table 1:** Hydrogen-bond geometry (Å, °)

*D*—H⋯*A*	*D*—H	H⋯*A*	*D*⋯*A*	*D*—H⋯*A*
C21—H21*A*⋯O1^i^	0.93	2.44	3.321 (3)	158

Symmetry code: (i) 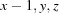.

**Fig. 1.**
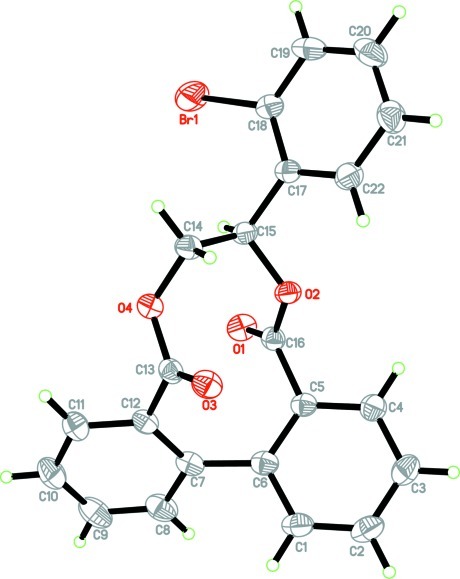
The molecular structure of the title compound showing 30% probability displacement ellipsoids for non-H atoms.

**Fig. 2.**
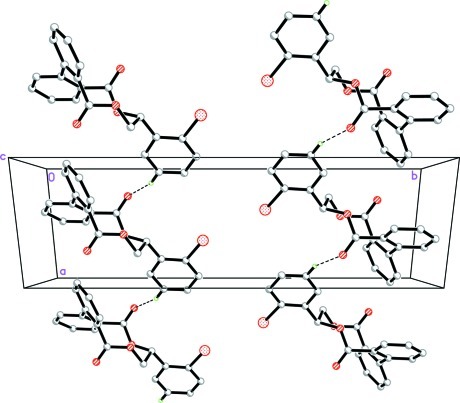
The crystal structure of the title compound, viewed along the c axis. H atoms not involved in hydrogen bonds (dashed lines) have been omitted for clarity.

## supplementary crystallographic information

### Comment

As part of our ongoing synthetic and structural studies of bis-lactones containing a biaryl motif (Wu *et al.*, 2012; Fun *et al.* 2012), we now report the structure of the title compound, (I).

In the title compound, Fig. 1, the bromo attached phenyl ring (C17-C22) inclines at dihedral angles of 23.87 (11) and 52.37 (11)° with the two benzene rings (C1-C6 and C7-C12), respectively. The two benzene rings form a dihedral angle of 49.08 (11)°. Bond lengths and angles are comparable to a related structure (Fun *et al.*, 2012).

In the crystal (Fig. 2), molecules are linked into one-dimensional chains propagating along the [100] direction *via* C21–H21A···O1 hydrogen bonds (Table 1).

### Experimental

The title compound was the major diastereoisomer of the sequential photoreaction products of 9,10-phenanthrenedione with 1-bromo-2-vinylbenzene. The compound was purified by flash column chromatography with ethyl acetate/petroleum ether (1:9) as eluents. Colourless blocks were obtained from slow evaporation of an acetone and petroleum ether solution (1:10).

### Refinement

All H atoms were positioned geometrically and refined using a riding model with C–H = 0.93-0.98 Å and *U*_iso_(H) = 1.2 *U*_eq_(C).

### Crystal data

**Table:**

C_22_H_15_BrO_4_	*F*(000) = 856
*M**_r_* = 423.25	*D*_x_ = 1.533 Mg m^−^^3^
Monoclinic, *P*2_1_/*c*	Mo *K*α radiation, λ = 0.71073 Å
Hall symbol: -P 2ybc	Cell parameters from 3936 reflections
*a* = 8.0436 (9) Å	θ = 2.7–25.1°
*b* = 21.775 (2) Å	µ = 2.27 mm^−^^1^
*c* = 12.8809 (11) Å	*T* = 296 K
β = 125.613 (5)°	Block, colourless
*V* = 1834.1 (3) Å^3^	0.41 × 0.23 × 0.17 mm
*Z* = 4	

### Data collection

**Table:**

Bruker SMART APEXII DUO CCD diffractometer	5372 independent reflections
Radiation source: fine-focus sealed tube	3377 reflections with *I* > 2σ(*I*)
Graphite monochromator	*R*_int_ = 0.028
φ and ω scans	θ_max_ = 30.1°, θ_min_ = 1.9°
Absorption correction: multi-scan (*SADABS*; Bruker, 2009)	*h* = −11→11
*T*_min_ = 0.456, *T*_max_ = 0.702	*k* = −30→26
17934 measured reflections	*l* = −18→17

### Refinement

**Table:**

Refinement on *F*^2^	Primary atom site location: structure-invariant direct methods
Least-squares matrix: full	Secondary atom site location: difference Fourier map
*R*[*F*^2^ > 2σ(*F*^2^)] = 0.039	Hydrogen site location: inferred from neighbouring sites
*wR*(*F*^2^) = 0.102	H-atom parameters constrained
*S* = 1.02	*w* = 1/[σ^2^(*F*_o_^2^) + (0.0405*P*)^2^ + 0.5554*P*] where *P* = (*F*_o_^2^ + 2*F*_c_^2^)/3
5372 reflections	(Δ/σ)_max_ = 0.001
244 parameters	Δρ_max_ = 0.50 e Å^−^^3^
0 restraints	Δρ_min_ = −0.64 e Å^−^^3^

### Special details

**Table:**

Geometry. All esds (except the esd in the dihedral angle between two l.s. planes) are estimated using the full covariance matrix. The cell esds are taken into account individually in the estimation of esds in distances, angles and torsion angles; correlations between esds in cell parameters are only used when they are defined by crystal symmetry. An approximate (isotropic) treatment of cell esds is used for estimating esds involving l.s. planes.
Refinement. Refinement of F^2^ against ALL reflections. The weighted R-factor wR and goodness of fit S are based on F^2^, conventional R-factors R are based on F, with F set to zero for negative F^2^. The threshold expression of F^2^ > 2sigma(F^2^) is used only for calculating R-factors(gt) etc. and is not relevant to the choice of reflections for refinement. R-factors based on F^2^ are statistically about twice as large as those based on F, and R- factors based on ALL data will be even larger.

### Fractional atomic coordinates and isotropic or equivalent isotropic displacement parameters (Å^2^)

**Table:**

	*x*	*y*	*z*	*U*_iso_*/*U*_eq_	
Br1	0.37032 (5)	0.921319 (11)	−0.01825 (3)	0.08022 (13)	
O1	0.7470 (2)	0.72827 (7)	0.16591 (14)	0.0531 (4)	
O2	0.4090 (2)	0.71970 (6)	0.07684 (12)	0.0418 (3)	
O3	0.2865 (2)	0.63389 (7)	−0.14111 (13)	0.0491 (3)	
O4	0.4228 (2)	0.72635 (6)	−0.13204 (13)	0.0458 (3)	
C1	0.6842 (4)	0.52668 (10)	0.1470 (2)	0.0553 (5)	
H1A	0.7364	0.4977	0.1199	0.066*	
C2	0.6125 (4)	0.50741 (10)	0.2159 (2)	0.0604 (6)	
H2A	0.6172	0.4660	0.2350	0.072*	
C3	0.5339 (4)	0.54928 (11)	0.2566 (2)	0.0584 (6)	
H3A	0.4837	0.5362	0.3022	0.070*	
C4	0.5300 (3)	0.61110 (10)	0.22937 (19)	0.0492 (5)	
H4A	0.4768	0.6396	0.2566	0.059*	
C5	0.6052 (3)	0.63062 (8)	0.16156 (17)	0.0401 (4)	
C6	0.6811 (3)	0.58824 (9)	0.11647 (18)	0.0446 (4)	
C7	0.7478 (3)	0.60440 (9)	0.03322 (19)	0.0452 (4)	
C8	0.9329 (4)	0.58139 (11)	0.0623 (2)	0.0610 (6)	
H8A	1.0154	0.5581	0.1359	0.073*	
C9	0.9969 (4)	0.59215 (13)	−0.0146 (3)	0.0683 (7)	
H9A	1.1197	0.5755	0.0068	0.082*	
C10	0.8806 (4)	0.62726 (12)	−0.1228 (2)	0.0621 (6)	
H10A	0.9231	0.6341	−0.1753	0.075*	
C11	0.6997 (3)	0.65224 (10)	−0.1529 (2)	0.0518 (5)	
H11A	0.6231	0.6775	−0.2242	0.062*	
C12	0.6307 (3)	0.64009 (9)	−0.07794 (18)	0.0414 (4)	
C13	0.4266 (3)	0.66420 (9)	−0.12071 (17)	0.0400 (4)	
C14	0.2663 (3)	0.75862 (10)	−0.13137 (19)	0.0484 (5)	
H14A	0.1485	0.7323	−0.1635	0.058*	
H14B	0.2226	0.7949	−0.1849	0.058*	
C15	0.3601 (3)	0.77669 (8)	0.00661 (18)	0.0412 (4)	
H15A	0.4844	0.8010	0.0408	0.049*	
C16	0.6025 (3)	0.69798 (9)	0.13718 (17)	0.0399 (4)	
C17	0.2098 (3)	0.81201 (8)	0.01800 (17)	0.0399 (4)	
C18	0.1965 (3)	0.87560 (9)	0.00813 (19)	0.0473 (5)	
C19	0.0585 (4)	0.90790 (11)	0.0178 (2)	0.0612 (6)	
H19A	0.0522	0.9505	0.0110	0.073*	
C20	−0.0691 (4)	0.87701 (13)	0.0375 (2)	0.0657 (7)	
H20A	−0.1608	0.8987	0.0453	0.079*	
C21	−0.0621 (3)	0.81435 (12)	0.0458 (2)	0.0611 (6)	
H21A	−0.1498	0.7934	0.0584	0.073*	
C22	0.0759 (3)	0.78209 (10)	0.0353 (2)	0.0504 (5)	
H22A	0.0786	0.7394	0.0401	0.061*	

### Atomic displacement parameters (Å^2^)

**Table:**

	*U*^11^	*U*^22^	*U*^33^	*U*^12^	*U*^13^	*U*^23^
Br1	0.0990 (2)	0.04491 (14)	0.1191 (3)	−0.00504 (13)	0.0762 (2)	0.00892 (13)
O1	0.0427 (8)	0.0506 (8)	0.0540 (8)	−0.0041 (7)	0.0214 (7)	−0.0010 (7)
O2	0.0430 (7)	0.0349 (6)	0.0469 (7)	0.0056 (5)	0.0258 (6)	0.0041 (5)
O3	0.0419 (8)	0.0497 (8)	0.0541 (8)	−0.0012 (6)	0.0271 (7)	−0.0058 (6)
O4	0.0504 (8)	0.0433 (7)	0.0495 (8)	0.0067 (6)	0.0324 (7)	0.0024 (6)
C1	0.0647 (14)	0.0422 (11)	0.0456 (11)	0.0113 (10)	0.0244 (11)	0.0006 (9)
C2	0.0753 (16)	0.0403 (11)	0.0461 (12)	−0.0002 (11)	0.0244 (12)	0.0042 (9)
C3	0.0655 (15)	0.0573 (13)	0.0467 (12)	−0.0009 (11)	0.0294 (12)	0.0096 (10)
C4	0.0524 (12)	0.0512 (12)	0.0400 (10)	0.0061 (9)	0.0246 (10)	0.0046 (9)
C5	0.0373 (10)	0.0400 (9)	0.0331 (9)	0.0041 (8)	0.0150 (8)	0.0010 (7)
C6	0.0409 (11)	0.0439 (11)	0.0367 (10)	0.0063 (8)	0.0156 (9)	−0.0002 (8)
C7	0.0397 (11)	0.0452 (10)	0.0446 (11)	0.0030 (9)	0.0211 (9)	−0.0070 (8)
C8	0.0466 (13)	0.0676 (15)	0.0569 (14)	0.0138 (11)	0.0235 (11)	−0.0041 (11)
C9	0.0454 (13)	0.0843 (18)	0.0758 (17)	0.0048 (12)	0.0355 (13)	−0.0193 (14)
C10	0.0550 (14)	0.0753 (16)	0.0704 (16)	−0.0072 (12)	0.0447 (13)	−0.0154 (13)
C11	0.0491 (12)	0.0590 (13)	0.0537 (12)	−0.0024 (10)	0.0336 (11)	−0.0057 (10)
C12	0.0390 (10)	0.0414 (10)	0.0428 (10)	−0.0011 (8)	0.0232 (9)	−0.0078 (8)
C13	0.0409 (11)	0.0452 (10)	0.0334 (9)	0.0034 (8)	0.0214 (8)	−0.0025 (8)
C14	0.0519 (12)	0.0461 (11)	0.0485 (11)	0.0128 (9)	0.0300 (10)	0.0054 (9)
C15	0.0440 (11)	0.0336 (9)	0.0465 (10)	0.0039 (8)	0.0267 (9)	0.0032 (8)
C16	0.0416 (11)	0.0418 (10)	0.0332 (9)	0.0026 (8)	0.0201 (9)	−0.0024 (7)
C17	0.0402 (10)	0.0376 (9)	0.0377 (9)	0.0035 (8)	0.0203 (9)	−0.0002 (7)
C18	0.0507 (12)	0.0382 (10)	0.0498 (11)	0.0029 (9)	0.0275 (10)	0.0022 (8)
C19	0.0630 (15)	0.0455 (12)	0.0664 (15)	0.0171 (11)	0.0328 (13)	0.0001 (10)
C20	0.0539 (14)	0.0805 (18)	0.0604 (14)	0.0230 (13)	0.0320 (12)	0.0015 (12)
C21	0.0482 (13)	0.0794 (17)	0.0607 (14)	0.0060 (12)	0.0345 (12)	0.0075 (12)
C22	0.0499 (12)	0.0460 (11)	0.0554 (12)	0.0019 (9)	0.0306 (11)	0.0048 (9)

### Geometric parameters (Å, º)

**Table:**

Br1—C18	1.901 (2)	C9—C10	1.372 (4)
O1—C16	1.193 (2)	C9—H9A	0.9300
O2—C16	1.357 (2)	C10—C11	1.381 (3)
O2—C15	1.449 (2)	C10—H10A	0.9300
O3—C13	1.196 (2)	C11—C12	1.391 (3)
O4—C13	1.360 (2)	C11—H11A	0.9300
O4—C14	1.446 (2)	C12—C13	1.490 (3)
C1—C2	1.377 (3)	C14—C15	1.524 (3)
C1—C6	1.393 (3)	C14—H14A	0.9700
C1—H1A	0.9300	C14—H14B	0.9700
C2—C3	1.375 (3)	C15—C17	1.511 (3)
C2—H2A	0.9300	C15—H15A	0.9800
C3—C4	1.387 (3)	C17—C22	1.383 (3)
C3—H3A	0.9300	C17—C18	1.389 (3)
C4—C5	1.387 (3)	C18—C19	1.380 (3)
C4—H4A	0.9300	C19—C20	1.368 (4)
C5—C6	1.405 (3)	C19—H19A	0.9300
C5—C16	1.497 (3)	C20—C21	1.367 (4)
C6—C7	1.494 (3)	C20—H20A	0.9300
C7—C8	1.400 (3)	C21—C22	1.385 (3)
C7—C12	1.404 (3)	C21—H21A	0.9300
C8—C9	1.377 (4)	C22—H22A	0.9300
C8—H8A	0.9300		
			
C16—O2—C15	117.39 (15)	C7—C12—C13	120.85 (17)
C13—O4—C14	116.62 (15)	O3—C13—O4	124.52 (17)
C2—C1—C6	122.0 (2)	O3—C13—C12	125.52 (18)
C2—C1—H1A	119.0	O4—C13—C12	109.95 (16)
C6—C1—H1A	119.0	O4—C14—C15	106.92 (16)
C3—C2—C1	120.2 (2)	O4—C14—H14A	110.3
C3—C2—H2A	119.9	C15—C14—H14A	110.3
C1—C2—H2A	119.9	O4—C14—H14B	110.3
C2—C3—C4	119.6 (2)	C15—C14—H14B	110.3
C2—C3—H3A	120.2	H14A—C14—H14B	108.6
C4—C3—H3A	120.2	O2—C15—C17	108.48 (15)
C3—C4—C5	120.2 (2)	O2—C15—C14	106.09 (15)
C3—C4—H4A	119.9	C17—C15—C14	111.35 (16)
C5—C4—H4A	119.9	O2—C15—H15A	110.3
C4—C5—C6	120.85 (18)	C17—C15—H15A	110.3
C4—C5—C16	118.26 (17)	C14—C15—H15A	110.3
C6—C5—C16	120.89 (17)	O1—C16—O2	124.79 (18)
C1—C6—C5	117.15 (19)	O1—C16—C5	125.73 (18)
C1—C6—C7	118.28 (18)	O2—C16—C5	109.48 (16)
C5—C6—C7	124.49 (18)	C22—C17—C18	117.32 (18)
C8—C7—C12	116.7 (2)	C22—C17—C15	121.22 (17)
C8—C7—C6	119.65 (19)	C18—C17—C15	121.44 (18)
C12—C7—C6	123.65 (17)	C19—C18—C17	121.5 (2)
C9—C8—C7	122.1 (2)	C19—C18—Br1	117.61 (17)
C9—C8—H8A	118.9	C17—C18—Br1	120.89 (15)
C7—C8—H8A	118.9	C20—C19—C18	119.7 (2)
C10—C9—C8	120.4 (2)	C20—C19—H19A	120.1
C10—C9—H9A	119.8	C18—C19—H19A	120.1
C8—C9—H9A	119.8	C21—C20—C19	120.2 (2)
C9—C10—C11	119.3 (2)	C21—C20—H20A	119.9
C9—C10—H10A	120.4	C19—C20—H20A	119.9
C11—C10—H10A	120.4	C20—C21—C22	119.8 (2)
C10—C11—C12	120.7 (2)	C20—C21—H21A	120.1
C10—C11—H11A	119.6	C22—C21—H21A	120.1
C12—C11—H11A	119.6	C17—C22—C21	121.3 (2)
C11—C12—C7	120.75 (18)	C17—C22—H22A	119.3
C11—C12—C13	118.37 (18)	C21—C22—H22A	119.3
			
C6—C1—C2—C3	0.2 (3)	C7—C12—C13—O3	−55.8 (3)
C1—C2—C3—C4	−0.9 (3)	C11—C12—C13—O4	−58.4 (2)
C2—C3—C4—C5	−0.1 (3)	C7—C12—C13—O4	123.48 (18)
C3—C4—C5—C6	1.8 (3)	C13—O4—C14—C15	92.45 (19)
C3—C4—C5—C16	−178.53 (18)	C16—O2—C15—C17	−145.27 (15)
C2—C1—C6—C5	1.4 (3)	C16—O2—C15—C14	95.00 (18)
C2—C1—C6—C7	−175.6 (2)	O4—C14—C15—O2	−63.25 (19)
C4—C5—C6—C1	−2.3 (3)	O4—C14—C15—C17	178.92 (15)
C16—C5—C6—C1	177.95 (18)	C15—O2—C16—O1	20.9 (3)
C4—C5—C6—C7	174.39 (18)	C15—O2—C16—C5	−159.12 (15)
C16—C5—C6—C7	−5.3 (3)	C4—C5—C16—O1	124.1 (2)
C1—C6—C7—C8	−49.6 (3)	C6—C5—C16—O1	−56.2 (3)
C5—C6—C7—C8	133.7 (2)	C4—C5—C16—O2	−55.9 (2)
C1—C6—C7—C12	127.8 (2)	C6—C5—C16—O2	123.83 (18)
C5—C6—C7—C12	−48.9 (3)	O2—C15—C17—C22	−28.2 (2)
C12—C7—C8—C9	−1.0 (3)	C14—C15—C17—C22	88.2 (2)
C6—C7—C8—C9	176.6 (2)	O2—C15—C17—C18	153.52 (17)
C7—C8—C9—C10	1.1 (4)	C14—C15—C17—C18	−90.1 (2)
C8—C9—C10—C11	0.7 (4)	C22—C17—C18—C19	1.3 (3)
C9—C10—C11—C12	−2.7 (4)	C15—C17—C18—C19	179.6 (2)
C10—C11—C12—C7	2.9 (3)	C22—C17—C18—Br1	−179.10 (15)
C10—C11—C12—C13	−175.23 (19)	C15—C17—C18—Br1	−0.8 (3)
C8—C7—C12—C11	−1.0 (3)	C17—C18—C19—C20	0.0 (4)
C6—C7—C12—C11	−178.45 (19)	Br1—C18—C19—C20	−179.65 (18)
C8—C7—C12—C13	177.03 (18)	C18—C19—C20—C21	−0.9 (4)
C6—C7—C12—C13	−0.4 (3)	C19—C20—C21—C22	0.6 (4)
C14—O4—C13—O3	19.0 (3)	C18—C17—C22—C21	−1.7 (3)
C14—O4—C13—C12	−160.36 (15)	C15—C17—C22—C21	−179.99 (19)
C11—C12—C13—O3	122.3 (2)	C20—C21—C22—C17	0.7 (3)

### Hydrogen-bond geometry (Å, º)

**Table:**

*D*—H···*A*	*D*—H	H···*A*	*D*···*A*	*D*—H···*A*
C21—H21*A*···O1^i^	0.93	2.44	3.321 (3)	158

Symmetry code: (i) *x*−1, *y*, *z*.
